# IGF2 and IGF1R identified as novel tip cell genes in primary microvascular endothelial cell monolayers

**DOI:** 10.1007/s10456-018-9627-4

**Published:** 2018-06-27

**Authors:** Marchien G. Dallinga, Bahar Yetkin-Arik, Richelle P. Kayser, Ilse M. C. Vogels, Patrycja Nowak-Sliwinska, Arjan W. Griffioen, Cornelis J. F. van Noorden, Ingeborg Klaassen, Reinier O. Schlingemann

**Affiliations:** 1 Ocular Angiogenesis Group, Departments of Ophthalmology and Medical Biology, Amsterdam University Medical Centers, Academic Medical Center, Amsterdam, The Netherlands; 2 School of Pharmaceutical Sciences, University of Geneva, Geneva, Switzerland; 3 Angiogenesis Laboratory, Department of Medical Oncology, Amsterdam University Medical Centers, VU University Medical Center, Amsterdam, The Netherlands; 4 Department of Genetic Toxicology and Cancer Biology, National Institute of Biology, Ljubljana, Slovenia; 5 Department of Ophthalmology, University of Lausanne, Jules-Gonin Eye Hospital, Fondation Asile des Aveugles, Lausanne, Switzerland; 6 Ocular Angiogenesis Group, Department of Medical Biology, Amsterdam University Medical Centers, Academic Medical Center, Meibergdreef 15, Room L3-154, 1105 AZ Amsterdam, The Netherlands

**Keywords:** Angiogenesis, Tip cells, CD34, IGF2, Endothelial cells, Cultured cells, Endothelial growth factors

## Abstract

Tip cells, the leading cells of angiogenic sprouts, were identified in cultures of human umbilical vein endothelial cells (HUVECs) by using CD34 as a marker. Here, we show that tip cells are also present in primary human microvascular endothelial cells (hMVECs), a more relevant endothelial cell type for angiogenesis. By means of flow cytometry, immunocytochemistry, and qPCR, it is shown that endothelial cell cultures contain a dynamic population of CD34^+^ cells with many hallmarks of tip cells, including filopodia-like extensions, elevated mRNA levels of known tip cell genes, and responsiveness to stimulation with VEGF and inhibition by DLL4. Furthermore, we demonstrate that our in vitro tip cell model can be exploited to investigate cellular and molecular mechanisms in tip cells and to discover novel targets for anti-angiogenesis therapy in patients. Small interfering RNA (siRNA) was used to knockdown gene expression of the known tip cell genes angiopoietin 2 (ANGPT2) and tyrosine kinase with immunoglobulin-like and EGF-like domains 1 (TIE1), which resulted in similar effects on tip cells and sprouting as compared to inhibition of tip cells in vivo. Finally, we identified two novel tip cell-specific genes in CD34^+^ tip cells in vitro: insulin-like growth factor 2 (IGF2) and IGF-1-receptor (IGF1R). Knockdown of these genes resulted in a significant decrease in the fraction of tip cells and in the extent of sprouting in vitro and in vivo. In conclusion, this study shows that by using our in vitro tip cell model, two novel essential tip cells genes are identified.

**Electronic supplementary material:**

The online version of this article (10.1007/s10456-018-9627-4) contains supplementary material, which is available to authorized users.

## Introduction

New blood vessel sprouting is led by tip cells, a transdifferentiated phenotype of endothelial cells induced by pro-angiogenic factors including vascular endothelial growth factor (VEGF) [1, 2]. In vivo, the tip cell phenotype is characterized by extension of filopodia, enhanced migratory propensity, and mitotic quiescence [1, 3]. Tip cells differ in various aspects from the more proximal, proliferating stalk cells and the maturing phalanx cells [2], and express a distinct set of genes [4, 5]. Until recently, progress in research on tip cells has been slow because of the need to use laboratory animals, as an in vitro model of tip cells was lacking. However, we have identified tip cells in monolayer human umbilical vein endothelial cell (HUVEC) cultures employing CD34 as a marker [6]. In vivo, CD34 is expressed throughout the body at the luminal side of endothelial cells of small blood vessels and umbilical veins and on filopodia of tip cells [7], but we have shown that in monolayers of HUVECs that have been passaged at least three times, approximately 10% of the cells express high levels of CD34. Moreover, these CD34-positive HUVECs have a distinct phenotype, with striking similarities when compared to tip cells in vivo, including CD34^+^ filopodia-like extensions, mitotic quiescence, and expression of tip cell specific genes [6, 8].

As validation of our in vitro model for tip cells, we investigated first whether CD34^+^ tip cells are also present in monolayer cultures of primary human microvascular endothelial cells (hMVECs), as angiogenesis in vivo is initiated in microvessels [9]. In addition, we studied the effects on CD34^+^ tip cells after silencing of two known tip cell genes, the growth factor angiopoietin-2 (*ANGPT2*) and the receptor tyrosine kinase with immunoglobulin-like and EGF-like domains 1 (*TIE1*), both of which were shown by us previously to have higher mRNA expression levels in CD34^+^ tip cells [6]. *ANGPT2* is involved in angiogenesis and interacts with the actin cytoskeleton to induce migration [10]. Silencing of ANGPT2 in mouse models of angiogenesis results in the absence of tip cells at the front of new vessel sprouts in mouse retinas [11]. TIE1 is an orphan receptor, which is expressed on tip cells and a subset of stalk cells, and which is involved in survival signaling in stalk cells [12].

Then, we explored the role in angiogenesis of two novel tip cell genes identified by us on the basis of differential expression in microarrays of CD34^+^ and CD34^−^ HUVECs, insulin-like growth factor 2 (IGF2), and insulin-like growth factor-1 receptor (IGF1R) (6). Both genes belong to the IGF family of growth factors, which consists of the ligands IGF1 and IGF2, the receptors IGF1R, IGF2R, and insulin receptor (INSR), and at least 7 IGF binding proteins (IGFBPs). IGF2 binds to and signals through IGF1R and the other IGF receptors. In earlier studies, knockdown of IGF2 and IGF1R inhibited angiogenesis in developing mouse retina and in zebrafish [13–15], but a specific role of these proteins in tip cells has not yet been reported. Here, we used our tip cell model to further characterize the role in angiogenesis of these novel tip cell genes.

## Materials and methods

### Cell cultures

Primary HUVECs were isolated from umbilical cords (obtained from the Department of Gynecology, Academic Medical Center, Amsterdam, The Netherlands), as described earlier [16], and grown in M199 basal medium (Gibco, Grand Island, NY, USA) supplemented with 10% heat inactivated human serum (obtained from the Department of Oncology, Academic Medical Center, Amsterdam, The Netherlands), 10% fetal bovine serum (Gibco), and 1% penicillin–streptomycin–glutamine (Gibco). HUVEC cultures were incubated with antibodies directed against CD31/PECAM-1 (1:100; eBioscience, Vienna, Austria) to check the purity of the endothelial cells. HMVECs, a kind gift of Dr. P. Koolwijk (VU University Medical Center, Amsterdam, The Netherlands), were cultured with 50% HUVEC medium and 50% EBM-2 medium (Lonza, Basel, Switzerland) and cells were characterized as previously described [17]. HUVECs and hMVECs were cultured in 2% gelatin-coated T75 culture flasks (Millipore, Billerica, MA, USA) at 37 °C and 5% CO_2_. Experiments were performed with confluent HUVECs at passage 3 and hMVECs at passage 9–10 of at least 3 different donors. Subjects gave informed consent for the use of tissues or serum, and samples were stored anonymously. Cells were treated with recombinant human VEGF-A (R&D Systems, Minneapolis, MN, USA), IGF2 (ProSpec, Rehovot, Israel), bFGF (Sanquin, Amsterdam, The Netherlands), or DLL4 (R&D Systems) as indicated.

### Immunocytochemistry

Cells were cultured on gelatin-coated coverslips (Thermo Scientific, South Logan, UT, USA) for 72 h when treated with siRNA or until confluent for spheroids and sorting experiments. Cells were fixed in freshly-made 4% paraformaldehyde in phosphate-buffered saline (PBS, Lonza) for 15 min at room temp, and then blocked in PBS containing 10% bovine serum albumin (BSA; Sigma-Aldrich, St. Louis, MO, USA) and 0.5% Triton X-100 (Sigma) for 1 h at room temperature. Next, cells were incubated with a primary antibody against CD34 (diluted 1:100, clone MD34.2; Sanquin) for 2 h and a secondary anti-mouse Alexa 488 antibody (Life Technologies, Carlsbad, CA, USA) and phalloidin (Life Technologies) to stain for F-actin for 1 h.

### DLL4 coating

Culture flasks were coated according to Harrington et al. [16] using 0.2% gelatin in PBS, with 1 µg/mL of either recombinant human DLL4 (R&D systems) or BSA for 24 h before the cells were seeded. After cells were cultured for 24 h, flow cytometric analysis was performed.

### Determination and selection of tip cells

For determining the percentage of tip cells, cells were harvested using TrypLE (Gibco), fixed in 4% paraformaldehyde in PBS for 15 min at room temp, and incubated with anti-CD34-phycoerythrin antibody (diluted 1:50; anti-CD34-PE; clone QBend-10, Thermo Scientific) for 30 min at room temperature. Cells were analyzed flow cytometrically using a FACSCalibur (Becton Dickinson, Franklin Lakes, NJ, USA) and FlowJo 6.4.7 software (Tree Star, San Carlos, CA, USA). The FITC channel was used to detect autofluorescence. Non-stained and non-treated cells were used as negative controls. For cell sorting experiments, cells were sorted on the basis of CD34 expression as detected  with anti-CD34-PE on a Sony SH800z cell sorter (Sony Biotechnology, Surrey, UK). CD34^−^ cells were cultured for 6 or 24 h, and then cells were fixed, stained, and analyzed using flow cytometry as described above.

### Apoptosis

Cellular apoptosis was assessed by measuring binding of annexin-V conjugated with FITC, following manufacturer’s instructions (Molecular Probes, catalog number: V13242, Eugene, OR, USA) in combination with staining for CD34 to determine  apoptosis in tip cells and non-tip cells.

### RNA isolation and quantitative PCR

Total RNA was isolated from cells using the TRIzol method according to the manufacturer’s instructions (Invitrogen, Carlsbad, CA, USA). An amount of 1 µg RNA was used for DNase I treatment (amplification grade; Invitrogen) and reverse transcribed into cDNA using the Maxima First Strand cDNA Synthesis Kit (Thermo Scientific). Real-time quantitative PCR (RT qPCR) was performed using a CFX96 real-time PCR detection system (Bio-Rad Laboratories, Hercules, CA, USA) as described previously [6]. Primer details are presented in Supplementary Table 1. NCBI BLAST confirmed the specificity of the primers. The presence of a single PCR product was verified by both the presence of a single melting temperature peak and detection of a single band of the expected size on agarose gels. Non-template controls were included to verify the method and the specificity of the primers. PCR products that did not show a single melting temperature peak were excluded from analysis. Ct values were converted to arbitrary absolute amounts (2^−*Ct*^ x 1E^12^) and expressed as fold change as compared to controls. Expression data were normalized to tyrosine 3-monooxygenase/tryptophan 5-monooxygenase activation protein zeta (YWHAZ) mRNA levels.

### siRNA knockdown

HUVECs and hMVECs were transfected with 25 nM of either a non-targeting small interfering RNA (siNT) or a gene-specific siRNA and 2.5 µg/mL DharmaFECT 1 transfection agent (Dharmacon, Lafayette, CO, USA). The cells were transfected for 6 h using the reversed transfection method according to the manufacturer’s instructions. Transfection efficiency was checked at the mRNA level and was considered acceptable when expression was reduced by at least 70% after 72 h.

### Spheroid-based sprouting assay

Spheroid experiments were performed with siRNA-transfected cells or cells that were sorted on the basis of CD34 expression. HUVECs transfected with siRNA were harvested after 48 h, and 750 cells per spheroid were seeded in methylcellulose (Sigma-Aldrich, Buchs, Switzerland) containing M199 medium and 2% human serum to allow spheroid formation [17, 18]. Cells sorted according to CD34 expression were immediately seeded in the same manner. After 18 h, the spheroids were embedded in collagen gels containing 0.5% human serum and, when indicated, treated with VEGF-A (25 ng/mL) or IGF2 (50 ng/mL), and were allowed to sprout for 24 h. Images were taken using a phase-contrast microscope and the number of sprouts and average sprout length per spheroid were analyzed using the Neuron-J plug-inn package of Image-J software [19]. Spheroid experiments were performed with HUVECs, since hMVECs did not sprout in this experimental setup.

### Statistics and data correction

To correct for differences between donors, data from flow cytometry and spheroid experiments were corrected using the Factor Correction program as described previously [20]. Statistical analysis was performed using a Student’s *t* tests.

### Chicken chorioallantoic membrane (CAM) assay

The anti-angiogenic efficacy of a custom si*IGF2* (Dharmacon) was tested in the CAM model [18, 21] via topical administration (each time 25 µl), between embryo development day (EDD) 7 and 8 once daily. Control eggs (blank) received 25 µl of HEPES (4-(2-hydroxyethyl)-1-piperazineethanesulfonic acid) buffer (Gibco) or 25 µl of non-targeting control siRNA (siNT) (SR-C2000-005; Eurogentec, Liege, Belgium) premixed with HEPES buffer and transfection reagent (DharmaFECT-1; Dharmacon). At EDD 9, the in ovo CAMs were visualized by means of FITC-dextran (20 kDa, 25 mg/mL; Sigma-Aldrich) epi-fluorescence angiography [22] and subsequently analyzed by an image-processing quantification method as described previously [23]. Briefly, on the basis of FITC-dextran fluorescence angiography, the skeleton overlay of the vascular network was placed on top of the vascular network, and branching points/mm^2^ were calculated.

### Zebrafish Morpholino experiments

Zebrafish experiments were performed with the approval of the Animal Ethics Committee of the University of Amsterdam and in compliance with the Association for Research in Vision and Ophthalmology (ARVO) statement for the Use of Animals in Ophthalmic and Vision Research. *Tg*(*Fli1a:eGFP*) transgenic zebrafish embryos were injected with Morpholino oligonucleotides against *IGF2*a or *IGF2*b that were designed and tested previously [15, 24]. For each gene, a 6-bp mismatch control was used, and a p53 Morpholino as a control for non-specific activation by Morpholino injection. After 24 and 30 h, the chorion was manually removed and zebrafish were mounted in 0.5% agarose gels and analyzed using confocal microscopy. Details of Morpholino sequences are shown in Supplementary Table 1.

## Results

### Human microvascular endothelial cell cultures contain CD34^+^ tip cells

**Fig. 1:**
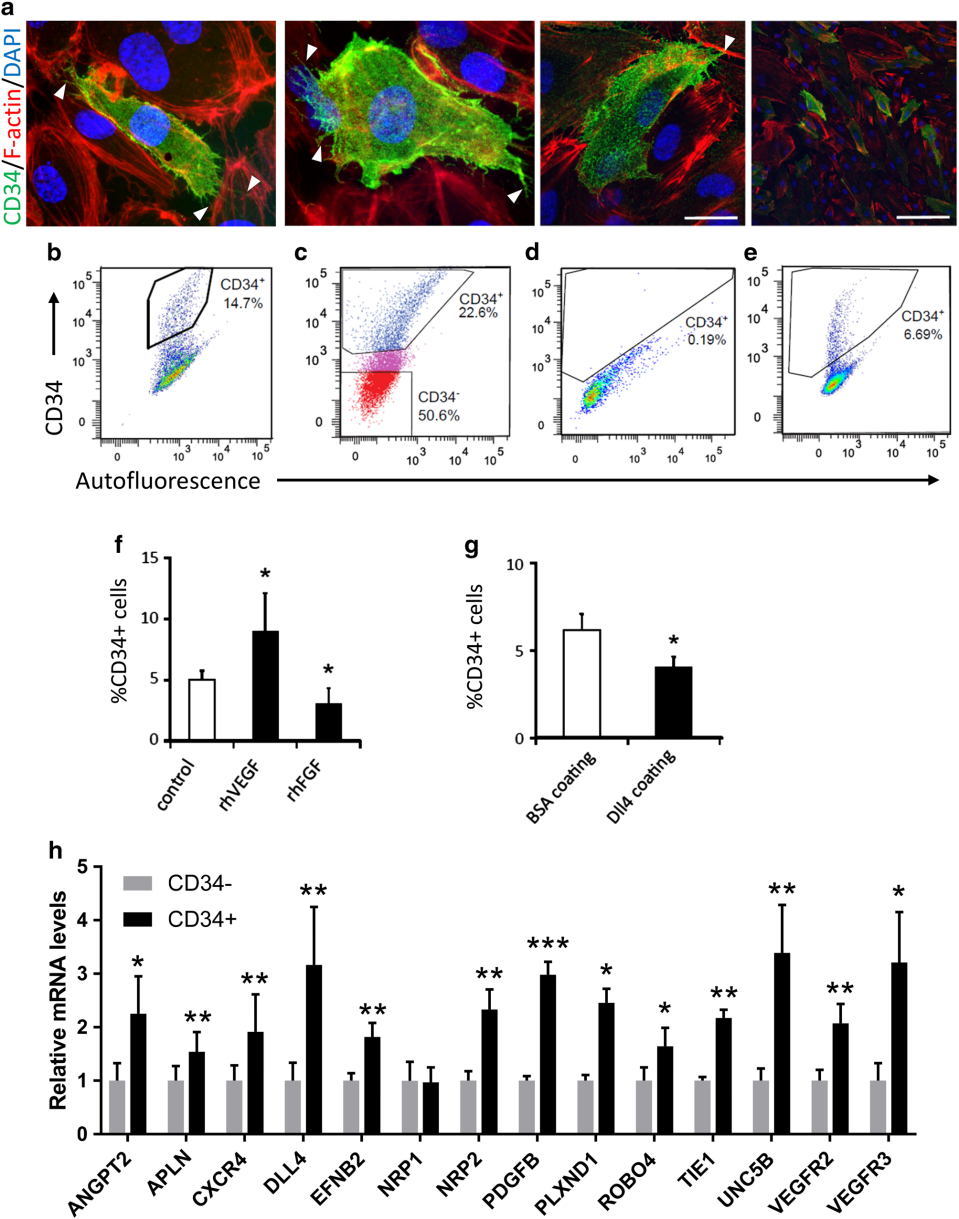
Human microvascular endothelial cell cultures contain CD34^+^ tip cells. **a** Identification of tip cells by staining with anti-CD34 (green), F-actin (phalloidin, red), and nuclei (DAPI, blue) in hMVECs. Representative examples are shown. Arrowheads indicate filopodia-like extrusions on CD34^+^ cells. Scale bar represents 25 µm (first 3 images) and 100 µm (last image). **b** HMVECs were analyzed for CD34 expression using  flow cytometry. **c**–**e** Re-expression of CD34 in hMVECs after cell sorting. CD34^−^ cells (shown in **c**) were cultured and CD34 expression was analyzed after 6 h (**d**) and 24 h (**e**). **f, g** The effect of exposure to VEGF or bFGF (**f**) and DLL4 (**g**) on the percentage of CD34^+^ tip cells. BSA was used as a control for DLL4. *p < 0.05 as compared to control. Data are shown as the mean ± standard deviation after factor correction (*n* = 3). **h** Fold change in mRNA expression levels of known tip cell genes in CD34^+^ hMVECs as compared to CD34^−^ hMVECs. Graph shows fold change of expression in CD34^+^ cells as compared to CD34^−^ cells after factor correction (*n* = 5). *p < 0.05, **p < 0.01, ***p < 0.001

We have previously identified CD34 as a marker for tip cells in monolayer cultures of HUVECs [6], which are of macrovascular origin. hMVECs are primary cells of microvascular origin, and derived from the endothelial cell types which generate new vessels in vivo, and may therefore be physiologically more relevant for studies of angiogenesis. To study whether tip cells are also present in monolayer cultures of hMVECs, we analyzed CD34 expression with flow cytometry and confocal microscopy, using anti-CD34 antibodies. This revealed a subpopulation of CD34^+^ hMVECs with filopodia-like extensions (Fig. 1a) of approximately 10% (Fig. 1b).

Freshly isolated HUVECs and hMVECs express CD34 on all cells [25, 26], but the percentage of CD34^+^ cells gradually tapers with increasing passage numbers until an equilibrium has been reached which is maintained for several passages. In HUVEC cultures, the equilibrium is reached at passage 3, whereas in hMVEC cultures the equilibrium is reached at passage 9. We examined whether CD34 expression in hMVECs either marks newly generated tip cells or identifies cells that have retained their phenotype since isolation. For this purpose, we sorted and cultured CD34^−^ hMVECs and found that between 6 and 24 h after the CD34^−^ cells were plated a new fraction of CD34^+^ cells appeared (Fig. 1c–e). This suggests that CD34^+^ cells develop de novo in CD34^−^ hMVEC cultures.

Next, we determined whether VEGF, DLL4, and bFGF changes tip cell differentiation, as measured by the percentage of tip cells in hMVECs, in a similar manner as was reported for tip cells in vivo. In the developing mouse retina, VEGF stimulates tip cell formation [1], whereas DLL4 inhibits the tip cell phenotype [27, 28] and bFGF induces proliferation of blood vessels [29]. In hMVEC cultures, VEGF significantly increased the percentage of CD34^+^ cells (Fig. 1f). In contrast, DLL4 and bFGF significantly decreased the percentage of CD34^+^ cells (Fig. 1f, g).

Analysis of mRNA expression of genes that have been associated with a tip cell phenotype in the developing mouse retina and zebrafish embryos [1, 4, 5, 30–38] showed that 13 out of the 14 genes have significantly higher expression levels in CD34^+^ than in CD34^−^ hMVECs (Fig. 1h).

These experiments show that CD34^+^ hMVECs are phenotypically and genotypically similar to tip cells in vivo.

### Knockdown of *ANGPT2* expression inhibits tip cell differentiation and sprouting

As a further validation of our in vitro tip cell model, we investigated whether knockdown of the expression of known tip cell genes affects the percentage of tip cells in a similar way as was reported in vivo. For this purpose, we selected *ANGPT2* and *TIE1*. Knockdown of *Angpt2* was reported to reduce the number of tip cells at the retinal sprouting front in the developing mouse retina [11, 39] and knockdown of the orphan receptor *Tie1* in mouse retinas resulted in stalk cell apoptosis [12]. Previously published microarray data (Geo Accession: GSE 34850) showed that *ANGPT2* and *TIE1* mRNA levels were significantly higher in CD34^+^ HUVECs than in CD34^−^ cells [6], which was confirmed for hMVECs by qPCR as well (Fig. 1h).

**Fig. 2:**
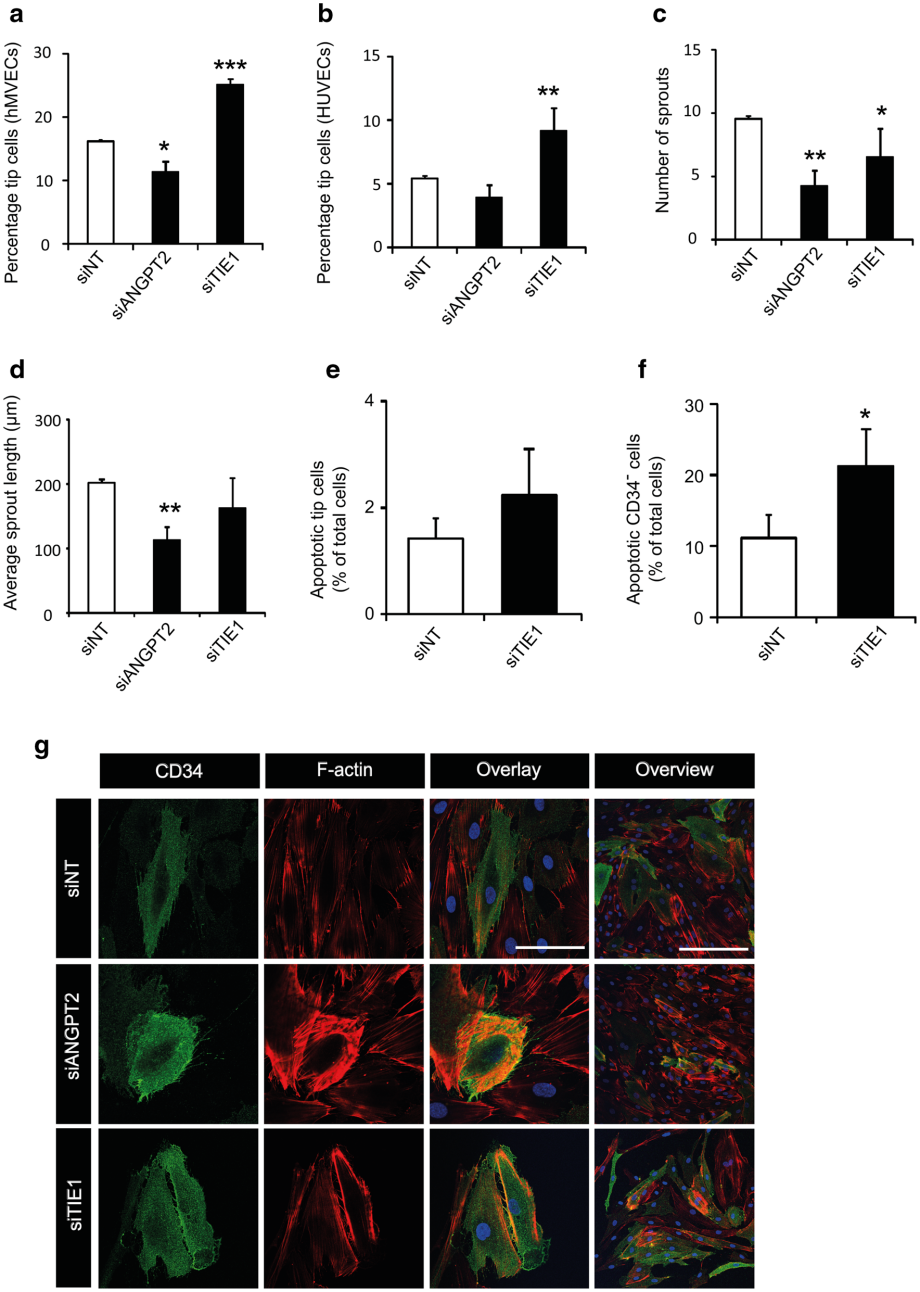
Effects of *ANGPT2* and *TIE1* knockdown on CD34^+^ tip cells and CD34^−^ non-tip cells in vitro. **a, b** Effect of knockdown of *ANGPT2* and *TIE1* expression on percentages of CD34^+^ tip cells. Bars show percentages of CD34^+^ hMVECs (**a**) and HUVECs (**b**) as detected by flow cytometry after treatment with non-targeting siRNA (siNT), *siANGPT2,* or *siTIE1*. **c, d** Quantification of numbers of sprouts (**c**) and average sprout length (**d**) of spheroids of HUVECs treated with siNT, *siANGPT2,* or *siTIE1*. **e, f** Percentages of apoptotic cells as detected by flow cytometry after treatment of HUVECs with siNT or *siTIE1*. Staining for CD34 was performed in combination with annexin-V staining of apoptotic CD34^+^ tip cells (**e**) and CD34^−^ cells (**f**). Data in **a–f** are shown as mean ± standard deviation after factor correction. *p < 0.05, **p < 0.01, ***p < 0.001 as compared to siNT (*n* = 3). **g** Analysis of CD34^+^ tip cell morphology after knockdown of *ANGPT2* and *TIE1* expression. Staining of CD34 (green), F-actin (phalloidin, red), and nuclei (DAPI, blue) in hMVECs and overlay images of higher and lower magnification. Note the cortical F-actin staining in the cells treated with siANGPT2. Scale bars represent 50 µm (first 3 columns) and 100 µm (last column)

Knockdown of *ANGPT2* expression by siRNA reduced the percentages of CD34^+^ tip cells significantly in hMVECs (Fig. 2a), but not in HUVECs (Fig. 2b). However, in the HUVEC spheroid-based sprouting model, sprout numbers and average sprout length were significantly decreased after knockdown of *ANGPT2* expression (Fig. 2c, d), and resulted in a disturbance of the actin cytoskeleton, as  the number of short radial stress fiber-like actin bundles was increased (Fig. 2g).

Knockdown of *TIE1* expression resulted in a significant increase in the CD34^+^ tip cell fraction in HUVECs (1.7-fold; Fig. 2a) and HMVECs (1.6-fold; Fig. 2b). The number of sprouts in the HUVEC spheroid model was marginally decreased (Fig. 2c), whereas sprout length was unaffected (Fig. 2d). Immunocytochemical staining of CD34 and F-actin showed that knockdown of *TIE1* expression did not result in morphological changes in HUVECs (Fig. 2g). After *TIE1* knockdown, apoptosis in CD34^−^ cells was higher (22.3%) than in siNT-treated HUVECs (11.1%), whereas the percentage of apoptotic tip cells was much lower (1.4%) with no difference as compared to siNT control (Fig. 2e, f). This indicates that at least part of the measured increase in tip cell percentage after *TIE1* knockdown was due to apoptosis of the CD34^−^ non-tip cells.

Together, we show that by using our in vitro tip cell model, we were able to reproduce observations reported on tip cells after *ANGPT2* and *TIE1* knockdown in vivo.

### Expression of IGF2 and IGF1R are essential for tip cell maintenance

**Fig. 3:**
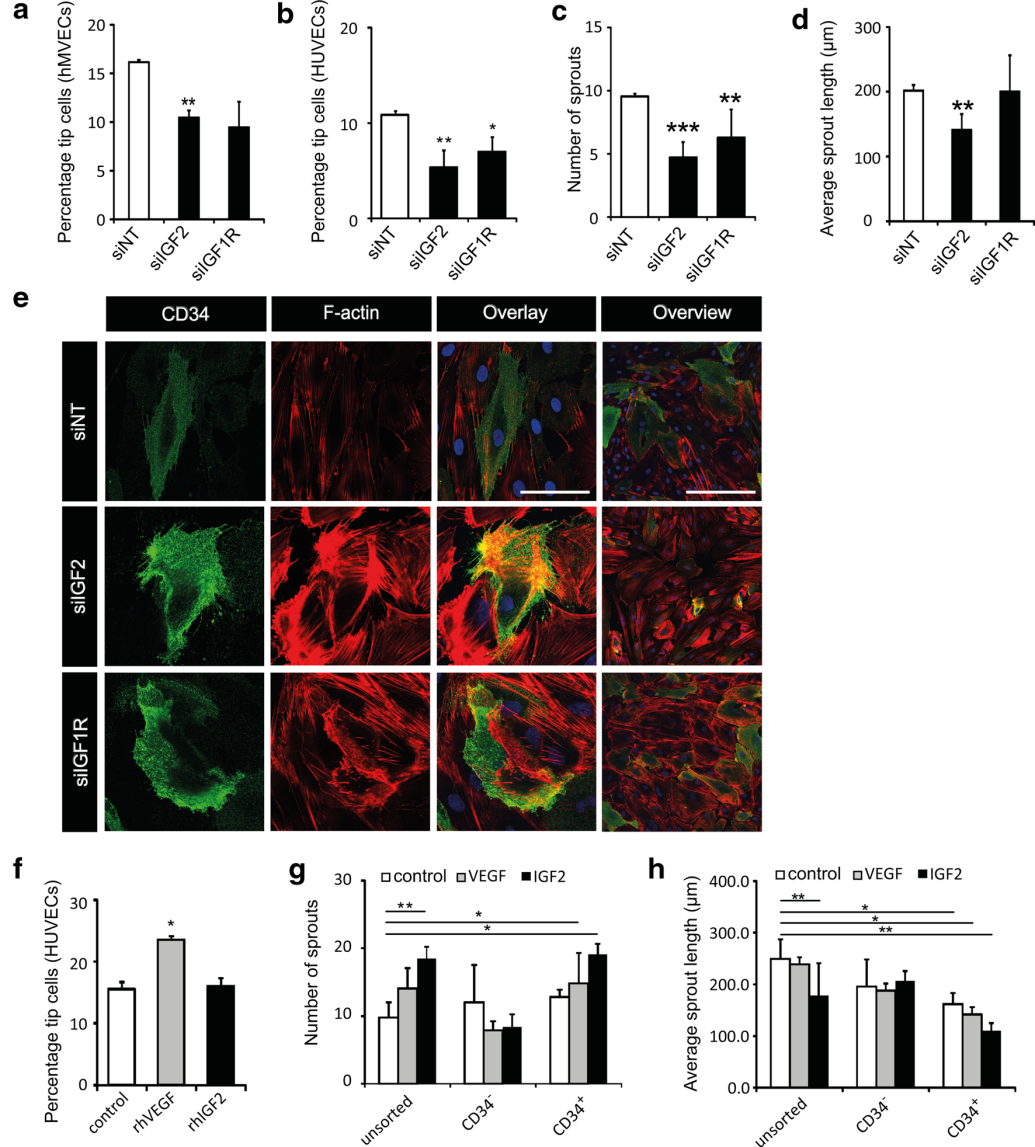
IGF2 and IGF1R are essential for CD34^+^ tip cell fate. **a, b** Effect of knockdown of *IGF2* and *IGF1R* expression on percentages of CD34^+^ tip cells. Bars show percentages of CD34^+^ hMVECs (**a**) and HUVECs (**b**) treated with siNT, *siIGF2*, or *siIGF1R* as detected by flow cytometry. **c, d** Quantification of numbers of sprouts (**c**) and average sprout length (**d**) of spheroids composed of HUVECs after treatment with siNT, *siIGF2*, or *siIGF1R*. **e** Analysis of CD34^+^ tip cell morphology after knockdown of *IGF2* and *IGF1R* expression. Staining of CD34 (green), F-actin (phalloidin, red), and nuclei (DAPI, blue) in hMVECs. Scale bars represent 50 µm (first 3 columns) and 100 µm (last column). **f** Effect of rhIGF2 on CD34^+^ HUVEC tip cell percentages. Bars shows CD34^+^ tip cells of HUVECs treated with either 25 ng/mL BSA, 25 ng/mL VEGF-A, or 50 ng/mL rhIGF2 as detected by flow cytometry. **g, h** Effects of VEGF and IGF2 on the number of sprouts (**g**) and average sprout length (**h**) in spheroids of CD34^+^ tip cells or CD34^−^ non-tip cells. Data in **a**–**d** and **f**–**h** are shown as mean ± standard deviation after factor correction. *p < 0.05, **p < 0.01, ***p < 0.001 as compared to control

Our microarray data of HUVECs showed that mRNA expression levels of *IGF2* were 45-fold higher in CD34^+^ cells as compared to CD34^−^ cells [6]. In addition, expression levels of *IGF1R*, a receptor for IGF2, were 2.1-fold higher in CD34^+^ cells [6]. IGF2 is known to stimulate angiogenesis in vitro, and increased IGF2 mRNA expression was found in vascular tufts in the retina of mice in the oxygen-induced retinopathy model [40] and in human vascular tumors such as hemangiomas [13, 40, 41]. To investigate whether IGF2 is specifically involved in tip cell fate, we performed knockdown of *IGF2* and *IGF1R* expression in hMVECs and HUVECs, which resulted in decreased percentages of CD34^+^ tip cells (Fig. 3a, b) and reduced numbers of sprouts per spheroid (Fig. 3c). Knockdown of expression of *IGF2* but not of *IGF1R* reduced sprout length (Fig. 3d). Knockdown of *IGF1R* expression increased the intensity of F-actin staining in hMVECs in all cells. Knockdown of *IGF2* seemed to increase the intensity of F-actin staining in all cells as well, but most strongly in CD34^+^ cells (Fig. 3e).

To investigate the effects of exogenous IGF2 on the percentage of tip cells and sprouting, we exposed HUVECs to IGF2 (50 ng/ml) in both models. Addition of IGF2 did not alter the CD34^+^ fraction in HUVEC cultures (Fig. 3f), but did increase the number of sprouts per spheroid (Fig. 3g, h). Next, we determined the effects of IGF2 on sprouting from spheroids composed of sorted CD34^−^ cells, CD34^+^ cells, and unsorted human microvascular endothelial cells (HMEC-1s). We used the immortalized cell line HMEC-1 for these experiments, as the timespan before appearance of de novo-generated CD34^+^ tip cells in CD34^−^ cultures is over 72 h in HMEC-1 cells, but only 24 h in hMVECs and HUVECs. Lack of expression of CD34 protein was confirmed by immunocytochemistry on HMEC-1 spheroids after completion of the experiment (data not shown). Similarly to spheroids of unsorted cells, in spheroids of CD34^+^ cells the number of sprouts was increased by VEGF and IGF2. In contrast, spheroids composed of CD34^−^ cells had less sprouts per spheroid and did not respond to VEGF or IGF2 (Fig. 3h, i). We performed an apoptosis assay to determine whether the effects observed on tip cell percentages and sprouting were caused by apoptosis (Supplementary Fig. 1). Treatment with siIGF2 resulted in higher (*p* < 0.05) percentages of apoptotic cells in the tip cell (10%) and CD34^−^ cell (19%) fractions, respectively, as compared to siNT (5 and 8%, respectively). Treatment of HUVECs with siIGF1R did not alter the percentage of apoptotic cells as compared to siNT.

**Fig. 4:**
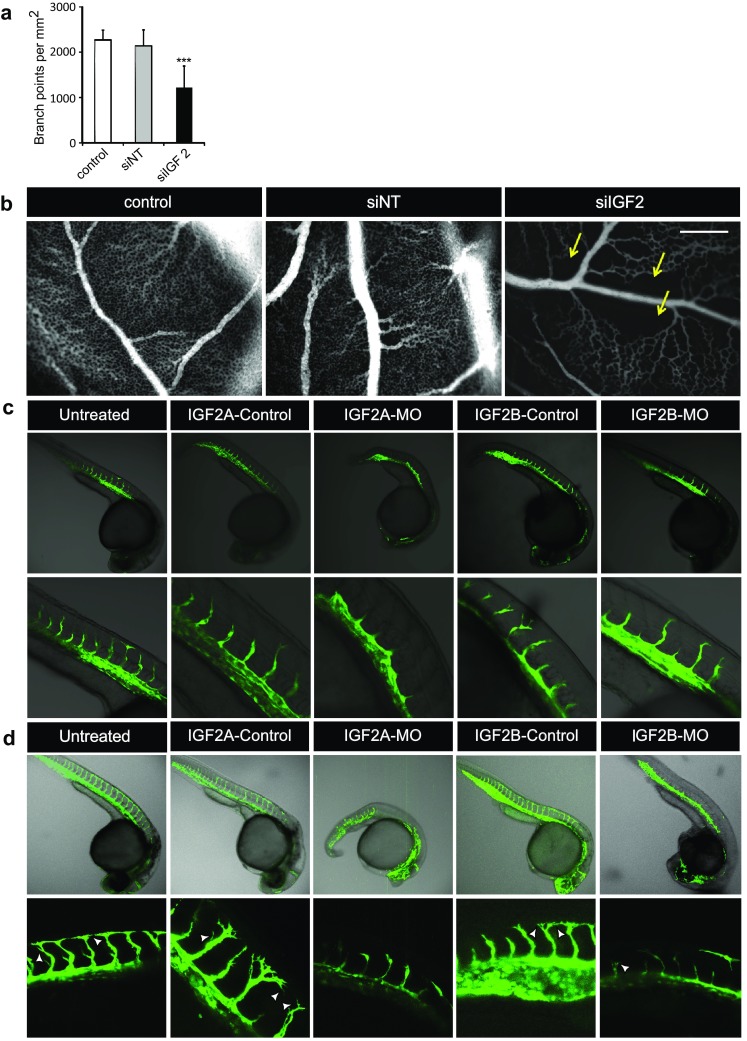
IGF2 is essential for sprouting in the CAM model and zebrafish embryos. **a** Quantification of number of branching points/mm^2^ comparing untreated membranes and membranes treated with siNT or with *siIGF2* in the CAM model. ***p < 0.001 as compared to control as well as siNT treatment. **b** Representative images of the vascular network in CAMs of chicks that were treated with siNT or *siIGF2* and untreated control. Arrows indicate non-vascularized areas in the vascular network. Scale bar represents 500 µm. **c, d** Representative images of *Tg*(*fli1a-eGFP*) zebrafish embryos at 24 h (**c**) and 30 h (**d**) after injection of either a Morpholino targeting *Igf2a* (IGF2A-MO) or *Igf2b* (IGF2B-MO) or a 6-bp mismatch control Morpholino for each gene (IGF2A-CON and IGF2B-CON, respectively). Untreated zebrafish embryos are shown as a control. Arrowheads indicate filopodia

To further investigate the role of IGF2 in angiogenesis, we knocked down IGF2 expression in vivo, using the developing CAM model in chicken embryos [23] and zebrafish embryos, respectively. In the CAM model, silencing of *IGF2* resulted in a twofold reduction in number of vascular branching points per mm^2^ (Fig. 4a, b), in larger avascular areas in between vessels, and in irregular vascular caliber (Fig. 4b). Gene silencing of the two zebrafish IGF2 isoforms (*IGF2A* and *IGF2B*) using Morpholinos in zebrafish embryos resulted in disturbed angiogenesis, albeit with a different phenotype for each isoform (Fig. 4c, d). *IGF2A* silencing caused delayed sprouting of intersegmental vessels (ISVs) after 24 h, and absence of filopodia at 30 h post-fertilization (Fig. 4c, d). Silencing of *IGF2B* resulted in chaotic sprouting of ISVs after 24 and 30 h with some ISVs developing slower than their neighbors, and reduced numbers of filopodia (Fig. 4c, d).

## Discussion

In the present study, we show that primary microvascular endothelial cells, hMVECs, contain a subset of CD34^+^ tip cells. This finding is similar to our earlier finding of CD34^+^ tip cells in macrovascular HUVEC cultures and immortalized endothelial cells such as HMEC-1 and RF24 [6], suggesting that the formation of tip cells in endothelial cell cultures is a general phenomenon. We found that knockdown of the expression of two known tip cell-specific genes, *ANGPT2* and *TIE1*, had the same effects on tip cell fate in vitro as has been reported on the basis of in vivo studies. In addition, we identified IGF2 and IGF1R to be highly expressed in CD34^+^ tip cells and we showed that these molecules are essential for tip cell fate and function in vitro and for angiogenesis in vivo. Together, these results further contribute to the acceptance of our in vitro tip cell model as a valid model. The use of our model will help to gain insight into novel cellular and molecular mechanisms that are of importance in tip cell biology.

### CD34 identifies tip cells in hMVEC cultures

CD34 is ubiquitously expressed on the luminal surface of endothelial cells of small blood vessels, but also has a striking presence on filopodia of tip cells in vivo [7, 42]. Although all endothelial cells express CD34 directly after isolation, after a certain number of passages CD34 expression is limited to a small fraction of cells, [25, 26] which we here identify as tip cells in vitro. Since CD34 expression re-appeared within 24 h in cultures of CD34^−^ hMVECs that were sorted by FACS, we conclude that these cells are generated de novo in endothelial cell cultures. Immunocytochemistry showed that CD34^+^ hMVECs in vitro have filopodia-like extensions that are positive for CD34, similarly to the extensions of tip cells in sprouting vascular fronts in mouse retina and in human colon carcinoma in vivo [1, 8, 43]. Stimulation of hMVECs with VEGF and DLL4 resulted in increased and decreased tip cell percentages, respectively, mimicking the regulatory effects on tip cells of these factors in vivo [1, 27, 28]. Finally, analysis of mRNA expression levels of genes known to be higher expressed in tip cells as compared to stalk cells in vivo demonstrated that these genes are also expressed at significantly higher levels in CD34^+^ cells than in CD34^−^ hMVECs.

Similar findings were previously demonstrated in our study of CD34^+^ cells in HUVECs [6]. In addition to the presently reported results in hMVECs, in HUVECs TNFα was shown to reduce the fraction of CD34^+^ tip cells, and CD34^+^ cells showed a higher capacity in cell migration and a much lower proliferation rate than CD34^−^ cells [6]. Genome-wide mRNA profiling analysis of CD34^+^ cells demonstrated enrichment for biological functions related to angiogenesis and migration, whereas CD34^−^ cells were enriched for functions related to proliferation. Furthermore, gene set enrichment analysis (GSEA) showed that our gene set and gene sets of other studies comprising the transcriptional profile of tip cells [4, 5, 16] correlated strongly.

Taken together, we conclude that the CD34^+^ fraction in endothelial cell cultures can be used to study what molecular mechanisms determine the formation of the tip cell phenotype, their behavior, and their gene expression. However, careful evaluation of experimental results is required to ascertain whether the outcome is due to positive effects on the CD34^+^ fraction, or rather by negative effects on the CD34^−^ fraction, or by a direct effect on CD34 itself. We have taken this into account in our present experiments. For example, bFGF reduced the percentage of CD34^+^ tip cells in hMVECs. Since bFGF is reported to stimulate endothelial cell proliferation during angiogenesis [29, 44] and CD34^+^ cells have a low proliferation rate, we interpreted the reduced percentage of CD34^+^ cells to be a result of increased proliferation of CD34^−^ cells, rather than of a direct downregulation of the numbers of CD34^+^ tip cells. However, the effect on cell proliferation should be further confirmed in experiments with FACS-sorted CD34^+^ and CD34^−^ cells exposed to bFGF. As another example, knockdown of *TIE1* increased the fraction of CD34^+^ cells. However, silencing of *TIE1* in developing mouse retinas causes increased apoptosis of stalk cells [12]. Similarly, in our study, knockdown of *TIE1* in vitro caused increased apoptosis of CD34^−^ but not of CD34^+^ cells. Therefore, we conclude that apoptosis of CD34^−^ cells upon knockdown of *TIE1* most likely contributes to or explains the observed increase in the fraction of CD34^+^ cells.

### Knockdown of *ANGPT2* inhibits endothelial cell sprouting

We studied the effects of *ANGPT2* and *TIE1* silencing on CD34^+^ tip cells to further validate our in vitro tip cell model as a tool to study genes enriched in tip cells, and to further study the role of these two genes in tip cell biology. Mice lacking *ANGPT2* have decreased numbers of tip cells at the sprouting front in the developing retinal vasculature, and show severely impaired angiogenesis [11, 39]. ANGPT2 binds to integrins that interact with the actin cytoskeleton, and thus may play a role in regulating cell migration [11, 45]. Our experiments in vitro revealed decreased tip cell percentages and decreased sprouting from spheroids upon knockdown of *ANGPT2*. Furthermore, the actin skeleton of cells treated with *siANGPT2* was disturbed. Since tip cells and stalk cells can switch phenotype [3], and as it has been hypothesized that the best-equipped cells become tip cells [3, 46], we hypothesize that knockdown of *ANGPT2* decreases migration of endothelial cells, which renders them unsuitable to become tip cells. Phenotype switching of neighboring non-tip cells requires migratory abilities to enable cells to reach the sprouting front [47]. Since neighboring cells also lack *ANGPT2* and are thus impaired in their migratory abilities, these cells are unable to take over the tip cell phenotype, which causes the tip cell fraction to decrease and sprouting to be reduced.

### IGF2 and IGF1R are essential for tip cell fate

We used our in vitro tip cell model as a tool to identify and characterize novel tip cell-specific genes. From our microarray data [6], that comprised more than 400 differentially expressed genes, we selected *IGF2*, a gene with one of the highest differences in mRNA levels in CD34^+^ cells as compared to CD34^−^ cells, and one of its receptors, *IGF1R*, which is also significantly higher expressed in CD34^+^ cells. On the basis of previous studies of knockdown of *IGF2* and *IGF1R* expression, it appeared that both proteins are important for angiogenesis in vivo and in vitro. This importance is reflected by reduced neovascularization in oxygen-deprived retinas, reduced mRNA expression in growing vascular tufts in developing mouse retinas, impaired angiogenesis in zebrafish embryos, and reduced sprouting in in vitro models of angiogenesis upon knockdown of *IGF2* and *IGF1R* [13–15]. Downstream pathways of IGF2 and IGF1R include pathways that are important for tip cells, such as phosphatidylinositol 3-kinases (PI3K), a kinase that promotes cell migration [48–50]. However, a specific role in tip cells has not yet been reported for either protein. We show here that IGF2 is essential for maintenance of the tip cell phenotype, as knockdown of IGF2 expression reduced the percentage of CD34^+^ tip cells, which could not be explained by tip cell-specific apoptosis, since the percentages of apoptotic cells were higher in CD34^−^ cells than in CD34^+^ tip cells (19 and 10%, respectively). IGF2 was also shown to be essential for angiogenesis, since knockdown of IGF2 expression reduced the number and length of sprouts in the spheroid assay. The role of IGF2 in angiogenesis was further supported by our in vivo experiments in the CAM assay and zebrafish, which show that sprouting in the absence of *IGF2* mRNA is chaotic and irregular.

Our experiments using spheroids composed of sorted CD34^+^ cells, CD34^−^ cells, or mixed populations of cells show that stimulation with IGF2 increased the number of sprouts in CD34^+^ spheroids and in spheroids composed of mixed populations, but not in CD34^−^ spheroids. This suggests that IGF2 mainly acts on tip cells, which is confirmed by the staining for F-actin: disturbances in the actin skeleton upon knockdown of *IGF2* occurred mainly in tip cells. On the other hand, stimulation with exogenous IGF2 did not increase the fraction of tip cells cultured in monolayers, suggesting that the effects on sprouting of exogenous IGF2 may be dependent on other growth factors present in the gel in which the spheroids grow. Thus, our results suggest that that IGF2 alone does not induce de novo tip cell formation, but that endogenous IGF2 is necessary to maintain the tip cell phenotype, probably in an autocrine fashion.

IGF1R binds all ligands of the IGF family of growth factors: IGF1, IGF2, and insulin (INS) [51]. IGF1 and INS also play a role in angiogenesis, as has been shown in vivo and in vitro [52–55]. In the present study, we showed that tip cell percentages and sprouting are reduced upon knockdown of *IGF1R*, and that the actin skeleton is disturbed in all cells. This further supports the essence of the IGF family of growth factors, including IGF2, in tip cell formation and sprouting angiogenesis. We will further explore the underlying molecular mechanisms in future experiments.

## Conclusions

In addition to the identification of tip cells in HUVECs, HMEC-1 cells, and RF24 cells, CD34 marks tip cells in cultures of hMVECs. This suggests that the existence of tip cells in endothelial cell cultures is a general phenomenon. Studying CD34^+^ tip cells in vitro can improve our understanding of tip cell biology by identifying proteins that are essential for tip cells, as was shown for ANGPT2 and TIE1. Finally, we provide evidence that IGF2 and IGF1R are novel tip cell proteins, and show that they are essential for maintenance of the tip cell phenotype.

## Electronic supplementary material

Below is the link to the electronic supplementary material.

**Supplementary Fig. 1 Increased apoptosis in CD34**^**+**^ **and CD34**^**-**^ **cells after IGF2 knockdown**. (**a-b**) Percentage of apoptotic CD34^+^ tip cells (**a**) and CD34^-^ cells (**b**) as detected by flow cytometry after treatment of HUVECs with siNT or *siIGF2* or *siIGF1R*. Labeling for CD34 was performed in combination with annexin-V staining. Data are shown as the mean ± standard deviation after factor correction. * *p* < 0.05. (EPS 59232 KB)

**Supplementary Fig. 2. Angiogenic sprouting in the spheroid-based sprouting model**. (a) Representative images of HMEC-1 spheroids of CD34^+^ cells after VEGF and IGF2 treatment as compared to untreated control. (b) Representative images of HUVEC spheroids treated with siNT, *siANGPT2* or *siTIE1*. (c) Representative images of HUVEC spheroids treated with siNT, *siIGF2 o*r *siIGF1R*. Scale bar represents 200 µm. (EPS 107216 KB)

**Supplementary Table 1. Primer and morpholino sequences used in this study**. Gene nomenclature, GenBank accession code, sequences of forward (Fw) and reverse (Rv) primers, size in base pairs (bp) and melting temperature (Tm) of the amplified product are indicated for each gene. For zebrafish experiments Morpholino sequences are indicated. For each isoform (IGF2a and IGF2b) two probes were used, adapted from reference 15 and 25 for probe 1 and 2, respectively. (PDF 77 KB)
