# Supplementary material for: IGF2 and IGF1R identified as novel tip cell genes in primary microvascular endothelial cell monolayers
Source: Angiogenesis. 2018 Jun 27;21(4):823–36. doi: 10.1007/s10456-018-9627-4 (PMC6208896; doi:10.1007/s10456-018-9627-4)
Supplement: Supplementary file 3 — Supplementary Table 1. Primer and morpholino sequences used in this study. Gene nomenclature, GenBank accession code, sequences of forward (Fw) and reverse (Rv) primers, size in base pairs (bp) and melting temperature (Tm) of the amplified product are indicated for each gene. For zebrafish experiments Morpholino sequences are indicated. For each isoform (IGF2a and IGF2b) two probes were used, adapted from reference 15 and 25 for probe 1 and 2, respectively. (PDF 77 KB) [file 10456_2018_9627_MOESM3_ESM.pdf]

Supplementary Table 1. Primer and morpholino sequences used in this study.
Gene nomenclature, GenBank accession code, sequences of forward (Fw) and reverse (Rv) primers, size in base pairs (bp) and melting temperature (Tm) of the amplified product are indicated for each gene. For zebrafish experiments morpholino sequences are indicated. For each isoform (IGF2a and IGF2b) two probes were used, adapted from reference 15 and 23 for probe 1 and 2, respectively.

PCR primers:

**Gene**	**GenBank**	**Fw**	**Rv**	**bp**	**Tm**
IGF2	NM_000612	CCTCGTGCTGCATTGCTGCT	CTTGCGGGCCTGCTGAAGTAGAA	115	86
IGF1R	NM_000875	GGAGCCCTGTGGTTGGATGTATG	TCATCTGGAAACGTCCGGTCGT	108	81
ANGPT2	NM_001147	GCAAAATAAGCAGCATCAGCCAAC	GCATCAAACCACCAGCCTCCT	115	76
TIE1	NM_005424	TGCAGCCCTCTGACAAGGACGA	CCTTCATCTTGCCCCCACTG	112	83
APLN	NM_017413	CCAGATGACAACCAGACGGACAG	GGCACCATTCCACCAAAAGATG	90	83
CD34	NM_001025109	GCCAGGTATTACAACTGGGTGTCCT	TCTGGTTGTCCACAGAGCCTTTGT	165	82
CXCR4	NM_003467	AACGGGGGACAGTGCCTGAA	TGAGCCCATTCTCCAGGTCAT	109	78
DLL4	NM_019074	AACGGGGGACAGTGCCTGAA	TGAGCCCATTCTCCAGGTCAT	151	86
EFNB2	NM_004093	GTTGGACAAGATGCAAGTTCTGCT	GCTGTTGCCGTCTGTGCTAGAA	147	80
NRP1	NM 003873	GCCTGCAACTTGGGAAACTGG	CCTTGGTTGGATGATGTGATCTGG	138	78
NRP2	NM 003872	GGAGCCCTGTGGTTGGATGTATG	TCATCTGGAAACGTCCGGTCGT	93	83
PDGFB	NM_002608	TCCAGGTGAGAAAGATCGAGATTG	CGTTTTGGCTCGCTGCTCCT	158	86
PLXND1	NM_015103	GACCCCGACACCCTACACATCT	CCTGCGCGATGACTGAAAGG	139	83
ROBO4	NM_019055	GCGTCTTCATAGATGCCTCATCAC	TGGCTGACCTCCATGTCTTCCAAC	121	85
UNC5B	NM_170744	CAGCCTAGATGCCCCCAACTCA	TCCCAGAGGTCCAGGATCACAC	129	83
VEGFR2	NM_002253	CCAGATGACAACCAGACGGACAG	GGCACCATTCCACCAAAAGATG	104	77
VEGFR3	NM_002020	GCCAGGTATTACAACTGGGTGTCCT	TCTGGTTGTCCACAGAGCCTTTGT	136	83

Morpholinos:

**Gene**	**GenBank**	**Probe 1**	**Probe 2**
IGF2a	NM_131433	CACAGAATACATGGTAATCATCCAT	GTTTGTGGAAAGCGACGTTTACTTT
IGF2b	NM_001001815	AATGATGTTTTAGTTGGTCCTCCAT	TAGTGAAGGTCGGATTAAGTCCCCT
IGF2a control		CAGAGAATAGATCGTAATGATCGAT	
IGF2b control		AATGATCTTTTACTTGCTCGTCGAT	
P53 control		CCTCTTACCTCAGTTACAATTTATA	
